# Epidemic Metastatic Parotiditis

**Published:** 1881-07-20

**Authors:** Charles H. Miller

**Affiliations:** Kansas


### EPIDEMIC METASTATIC PAROTIDITIS.

BY CHARLES H. MILLER, M. D., OF KANSAS.

There is an epidemic of mumps prevailing at the present time throughout the greater portion of the United States. This epidemic has peculiar features, one of which is its metastatic tendency to the testicles, both of which are most frequently affected. As this metastasis is in dispute among medical authorities, some contending that it takes place but rarely, if at all, and then only from exposure to cold; and others expressing their utter contempt for the transmission by an absolute silence on the subject, it is important to demonstrate by clinical experience, just what the truth of the matter is.

In an article on this subject in the Medical Review, of New York, for April 30th ult., and in another in Medical and Surgical Reporter, of Philadelphia, for May 7th ult., I have attempted to prove, with the meagre materials before me at the time, that the epidemic now prevailing is distinguished from the ordinary epidemics of mumps, from the fact that metastasis occurs in nine-tenths of all cases, in spite of all the most stringent precautions against cold, in warm weather as well as cold. There seems to be an inherent disposition on the part of the epidemic to seize the testicles, whether the patient remains in bed or is up and about. Females, as a rule, escaping a metastasis.

This form of mumps I have termed "Epidemic Metastatic Parotiditis," portraying its distinguishing features and endeavoring to rob it of the terrors it was generally supposed to possess, since the epidemic is a mild one, no deaths having occurred either directly or indirectly from the disease, although the winter just passed has been unusually severe for all diseases.

I call attention to this matter because I would like to hear from your readers where such epidemics prevail, and whether or not they partake of the character mentioned. By giving this publicity, therefore, you will oblige not only me but your readers also, perhaps.

